# Combination of ester biosynthesis and ω-oxidation for production of mono-ethyl dicarboxylic acids and di-ethyl esters in a whole-cell biocatalytic setup with *Escherichia coli*

**DOI:** 10.1186/s12934-017-0803-9

**Published:** 2017-11-02

**Authors:** Youri M. van Nuland, Gerrit Eggink, Ruud A. Weusthuis

**Affiliations:** 1 Bioprocess Engineering, Wageningen University and Research, Wageningen, Netherlands; 2 Biobased Products, Wageningen University and Research, Wageningen, Netherlands

**Keywords:** Whole-cell biocatalysis, α,ω-dicarboxylic acids, Adipic acid, Esters, Monooxygenases

## Abstract

**Background:**

Medium chain length (C6–C12) α,ω-dicarboxylic acids (DCAs) and corresponding esters are important building blocks for the polymer industry. For DCAs of 12 carbon atoms and longer, a sustainable process based on monooxygenase catalyzed ω-oxidation of fatty-acids has been realized. For medium-chain DCAs with a shorter chain length however, such a process has not been developed yet, since monooxygenases poorly ω-oxidize medium-chain fatty acids (MCFAs). On the contrary, esterified MCFAs are ω-oxidized well by the AlkBGTHJ proteins from Pseudomonas putida GPo1.

**Results:**

We show that MCFAs can be efficiently esterified and subsequently ω-oxidized in vivo. We combined ethyl ester synthesis and ω-oxidation in one-pot, whole-cell biocatalysis in* Escherichia coli*. Ethyl ester production was achieved by applying acyl-CoA ligase AlkK and an alcohol acyltransferase, either AtfA or Eeb1.* E. coli* expressing these proteins in combination with the ω-oxidation pathway consisting of AlkBGTHJ, produced mono-ethyl DCAs directly from C6, C8 and C9 fatty acids. The highest molar yield was 0.75, for mono-ethyl azelate production from nonanoic acid. Furthermore, di-ethyl esters were produced. Diethyl suberate was produced most among the di-ethyl esters, with a molar yield of 0.24 from octanoic acid.

**Conclusion:**

The results indicate that esterification of MCFAs and subsequent ω-oxidation to mono-ethyl DCAs via whole-cell biocatalysis is possible. This process could be the first step towards sustainable production of medium-chain DCAs and medium-chain di-ethyl esters.

**Electronic supplementary material:**

The online version of this article (10.1186/s12934-017-0803-9) contains supplementary material, which is available to authorized users.

## Background

α,ω-dicarboxylic acids (DCAs) of medium chain length (C6–C12) and corresponding esters constitute an important share of building blocks for the polymer industry. However, their production from petrochemical resources using organic chemistry methods is associated with high gross energy requirements and severe greenhouse gas emissions [1]. These adverse effects have directed research to alternative, more sustainable production processes. In a promising alternative route, fatty acids or their methyl esters are converted by microbial cells expressing an alkane 1-monooxygenase, sometimes assisted by an alcohol oxidase or dehydrogenase. This route has resulted in high titers of DCAs or mono-esterified DCAs (MEDAs) [2–4].

The AlkBGTL system from *Pseudomonas putida* GPo1 is especially suitable for the ω-oxidation of esterified medium chain length fatty acids [5]. Its main product is the ω-alcohol, and the ω-aldehyde and carboxylic acid are formed to a limited extent. Recently, we have expanded the AlkBGTL system with alcohol dehydrogenase AlkJ and aldehyde dehydrogenase AlkH in *E. coli*, resulting in the exclusive and efficient production of MEDAs from esterified fatty acids [6]. However, the AlkBGTLHJ system is not efficient in ω-oxidizing non-esterified medium-chain fatty acids. So esterification of medium-chain fatty acids in vitro by organic chemical methods prior to the oxidation step is required, adding costs to the overall process.

The solution is straightforward: if fatty acids have to be used directly, the biocatalyst has to esterify the fatty acids first in vivo. In vivo esterification of fatty acids to ethyl esters for the sake of biodiesel production in *E. coli* has been shown before [7, 8]. This conversion is accomplished in two steps. First the fatty acid is converted to acyl-CoA by a fatty acid CoA ligase, after which the coenzyme A moiety is exchanged with an alcohol by an alcohol:acyltransferase. In earlier studies aiming at ethyl ester production in *E. coli*, the native fatty-acid CoA ligase was used. For the alcohol:acyltransferase reaction AtfA and Eeb1 have been applied [7–9]. AtfA is known for its broad substrate scope, and can even synthesize wax di-esters [10–12]. Eeb1 uses ethanol and medium-chain acyl-CoA as substrates [9].

**Fig. 1:**
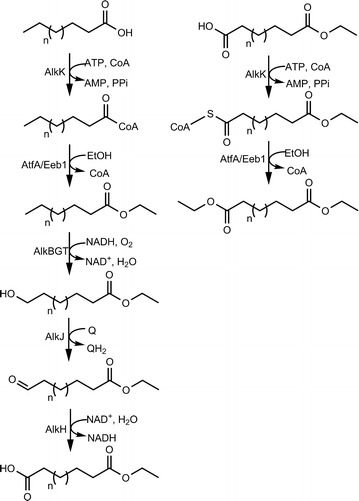
Proposed biocatalytic pathway. **a** Biocatalytic production of mono-ethyl dicarboxylic acids from fatty acids. **b** further conversion of mono-ethyl dicarboxylic acids to α,ω-diethyl esters

In this study, we investigated whether MEDAs can be produced directly from fatty acids. Two modules were used: an ω-oxidation module consisting of AlkBGTHJL and an esterification module consisting of an acyl-CoA ligase and an alcohol:acyltransferase. AlkK from *Pseudomonas putida* GPo1 was selected for acyl-CoA production from fatty acids, because we expect it to have the same broad substrate specificity as the enzymes encoded by AlkBGTLHJ. AlkK has been shown to synthesize octanoyl-CoA from octanoic acid [13, 14], but has not been tested with other medium-chain substrates to our knowledge. AtfA or Eeb1were selected to convert the generated acyl-CoA subsequently into ethyl esters [7, 9]. Together they realize the pathway depicted in Fig. 1a.

We also investigated if the esterification module is able to esterify the MEDAs into di-esterified DCAs (DEDAs, Fig. 1b). DEDAs are less soluble in water which would be advantageous for product removal from the broth. Furthermore, DEDAs are beneficial for polymerization processes, since in the polymerization process volatile alcohols are released instead of water, shifting the equilibrium of the reaction to polymer formation. Medium-chain DEDAs are also good building blocks for lipase-catalyzed polymerizations [15].

## Methods

### Chemicals

The following chemicals were purchased with the highest purity available: hexanoic acid, octanoic acid, nonanoic acid, methyl nonanoate, ethyl nonanoate, ethyl hydrogen adipate, ethyl hydrogen pimelate, diethyl azelate and diethyl sebacate from Sigma; ethyl hydrogen suberate, ethyl hydrogen sebacate and diethyl suberate from Alfa Aesar; ethyl hydrogen azelate from TRC, diethyl adipate from Acros Organics; diethyl pimelate from Merck; dimethyl octadecanedioate from TCI; 0.2 M TMSH in MeOH from Macherey–Nagel; Coenzyme A from VWR and ATP from GE Healthcare.

### Strains and plasmids

For cloning purposes, *E. coli* competent TOP10 (Invitrogen™) cells were used. For conversion studies, *E. coli* competent T7 Express (New England Biolabs^®^) cells were used. An overview of plasmids and strains used is shown in  Table 1.

**Table 1:** Plasmids used in this study

Plasmid	Description	Reference
pUC57-*alkKcodopt*	Codon optimized *alkK* in pUC57	This study
pUC57-*atfAcodopt*	Codon optimized *atfA* in pUC57	This study
pGEc47	Contains genes necessary for growth on alkanes (*alkBFGHJKL* and *alkST*) in the broad-host-range vector pLAFR1	[16]
pET-Duet-*alkK*	Codon optimized *alkK* under control of T7 promoter in MCSI of pET-Duet	This study
pCOM10-*alkL*	Contains *alkL* in pCOM10, a broad host range alkane responsive vector	[17]
pBGTHJKL-*atfA*	Complete *alk*-operon; codon optimized *atfA* under control of separate P_alkB_	This study
pBGTHJKL-*eeb1*	Complete *alk*-operon; *eeb1* under control of separate P_alkB_	This study
pE	*alkK* and *alkL* under control of P_alkB_; codon optimized *atfA* under control of separate P_alkB_ in pCOM10	This study
p*alkKL*	*alkK* and *alkL* under control of P_alkB_ in pCOM10	This study
pSTL	*alkTL* in pCOM10	[5]
pBGTHJL	*alkBGTHJL* genes in pCOM10	[6]
pE-II	pE with pBR322 ori, Amp^R^	This study

### Construction of vectors

Construction of vectors is shown in the Additional file 1: Table S1.

### Qualitative AlkK assay

*E. coli* carrying pET-Duet-*alkK* or pET-Duet (empty vector control) was grown overnight in LB containing 100 µg/mL ampicillin, at 30 °C, 250 rpm. Of this culture, 250 µL was used to inoculate 50 mL of the same medium. Expression of AlkK was started at an OD_600nm_ of 0.3 with the addition of 0.4 mM of IPTG. This culture was incubated overnight at 20 °C. Cells were harvested and resuspended in a buffer consisting of 25 mM Tris pH 7.5, 2.5 mM EDTA and 1% Triton-X100. Cells were then lysed with lysozyme and DNase I. The resulting mixture was centrifuged at 20,000×*g* for 15 min. The supernatant was loaded on a 10 kDa spin column to concentrate the protein. About 450 µg of this concentrate was added to a 5 min pre-incubated assay mixture consisting of 200 mM Tris pH 7.5, 12 mM MgCl_2_, 10 mM ATP, 1 mM CoA, and 2 mM octanoic acid or 2 mM ethyl hydrogen suberate. Reactions were carried out at 30 °C, and stopped by addition of 1:1 CHCl_3_:MeOH, brief vortexing and transferring the mixture to liquid nitrogen. The aqueous phase was analyzed with LC–MS/MS with a Waters BEH C8 column coupled to an LCQ-Fleet mass spectrometer.

### Whole-cell conversions and GC analysis

Cultivation, conversions and GC analysis were carried out as described before [5], except that induction with DCPK and the conversion were done at 30 °C. The substrates were added to 1 mM, from a concentrated stock in ethanol, to give a final concentration of ethanol of 2.5%. GC analysis of ω-acids was performed after derivatization with TMSH.

## Results

The ω-oxidation module system has been successfully tested before [6]. This system was expanded with AlkK and AtfA to realize the formation of esters. First we tested if AlkK and AtfA were functionally expressed and able to esterify both fatty acids and mono-ethyl dicarboxylic acids.

### Expression of AlkK

**Fig. 2:**
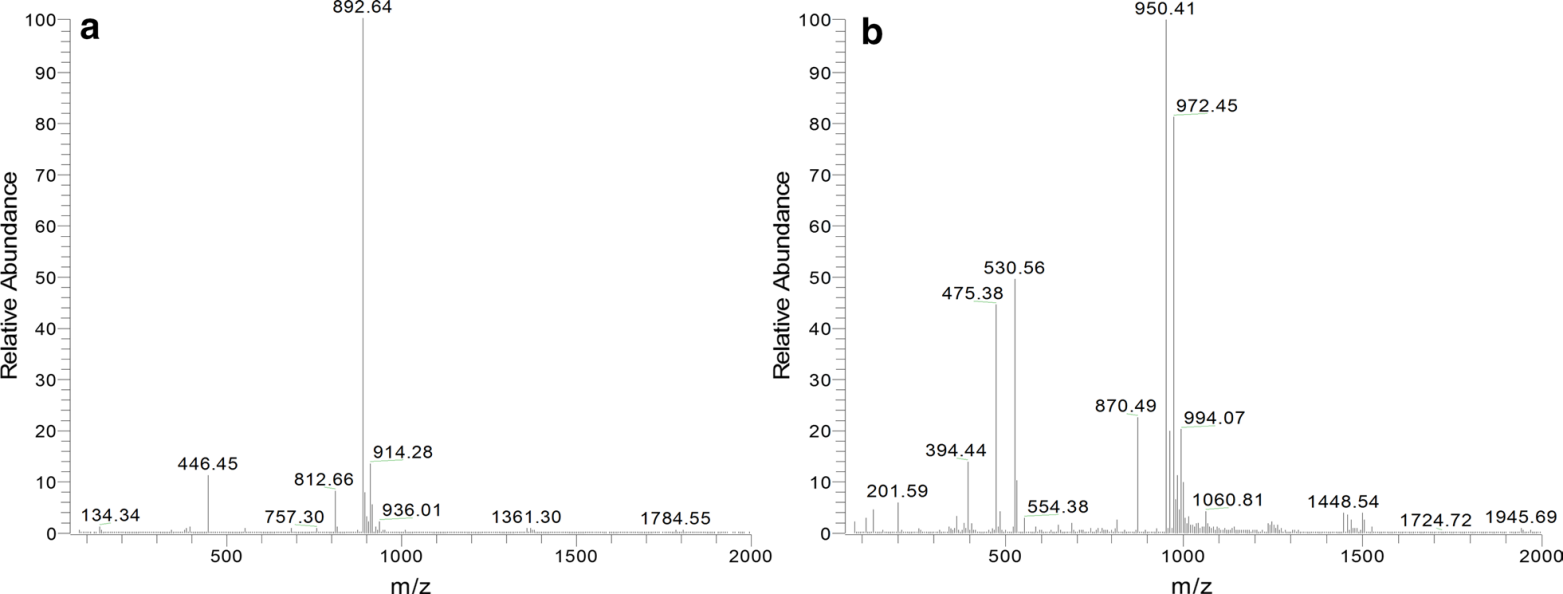
Mass spectra of products in assays with AlkK. **a** mass spectrum of the formed product in incubations with cell free extract of *E. coli* pET-Duet-*alkK* and octanoic acid. The m/z of 892 corresponds to octanoyl-CoA. **b** mass spectrum of the formed product in incubations with cell free extract of *E. coli* pET-Duet-*alkK* and mono-ethyl suberate. The m/z of 950 corresponds to the CoA-ester of mono-ethyl suberate. The M^−^+22*n peaks indicate sodium adducts

AlkK activity was determined by following the synthesis of the CoA esters from fatty acids and mono-ethyl diacids. The AlkK assay was performed qualitatively, since peak resolutions did not allow quantification. We tested functionality of AlkK in a cell-free extract assay, using octanoic acid, CoA and ATP in the assay mixture. The cell-free extracts were prepared from *E. coli* pET-Duet-*alkK* and *E. coli* pET-Duet (empty vector control). LC–MS analysis indicated that a product with an m/z of 892 was formed, see Fig. 2a. This mass corresponds to the expected mass of octanoyl-CoA.

In a negative control experiment we added cell-free extract of *E. coli* carrying the empty vector. In this assay only trace amounts of octanoyl-CoA were detected, indicating that the formed octanoyl-CoA in the cell-free extract from *E. coli* pET-Duet-*alkK* was formed by AlkK. We repeated the assay, with mono-ethyl suberate as substrate, to check whether AlkK also has the ability to generate the CoA-ester of mono-ethyl suberate. A product with m/z of 950 appeared (see Fig. 2b), which corresponds to mass for the CoA ester of ethyl hydrogen suberate. This implies that AlkK can also use mono-ethyl suberate as substrate.

### Combined expression of AlkK and AtfA

CoA esters of mono-ethyl dicarboxylic acids are not available. We therefore tested AtfA activity by using a combined assay with whole-cells expressing both AlkK and AtfA. The tests were performed with induced, resting *E. coli* pE cells. The same setup was tested with *E. coli* p*alkKL*, which served as negative control. Octanoic acid and nonanoic acid were applied as the substrate, added to 1 mM from a concentrated ethanol stock. This resulted in a final ethanol concentration of 2.5% v/v. Results are shown in Table 2.

**Table 2:** Whole-cell conversions of fatty acids (1 mM) and MEDAs (5 mM) into ethyl esters and di-esters

	*E. coli* pE	*E. coli* p*alkKL*
	Ethyl ester/di-ethyl ester concn (mM)	Ethyl ester/di-ethyl ester concn
Octanoic acid	0.72 ± 0.03	ND
Nonanoic acid	0.82 ± 0.12	ND
Mono-ethyl adipate (C6)	0.17 ± 0.00	< 0.01 mM
Mono-ethyl pimelate (C7)	0.12 ± 0.00	0.03 ± 0.00 mM
Mono-ethyl suberate (C8)	0.19 ± 0.02	0.02 ± 0.00 mM
Mono-ethyl azelate (C9)	0.05 ± 0.00	− 0.02 ± 0.00 mM
Mono-ethyl sebacate (C10)	0.06 ± 0.01	− 0.01 ± 0.00 mM
Mono-methyl azelate (C9)	0.50 ± 0.03^a^	NT

Incubation was carried out with 1.0 g_cdw_/L, and lasted for 2 h with fatty acids, 3 h for mono-ethyl dicarboxylic acids

*ND* not detected, *NT* not tested

^a^Detected product was methyl-ethyl azelate

AtfA was functionally expressed, because ethyl octanoate was produced by resting cells expressing both AlkK and AtfA. Ethyl octanoate was not detected in cells expressing only AlkK and AlkL from the p*alkKL* plasmid. Comparable results were achieved with nonanoic acid as substrate. The maximum product titer of 0.82 mM was reached with this substrate. A small gap in the C-balance was observed (0.18 of 1 mM added substrate), which was likely caused by evaporation of the volatile product and/or β-oxidation.

### Testing the AlkK-AtfA esterification module for esterification of MEDAs

The same test was performed using 5 mM of C6–C10 MEDA instead of fatty acids using *E. coli* pE. Di-ethyl esters were produced from all chain lengths. The titers were rather low, and 4% of the MEDA was converted to di-ethyl ester at most. Especially with C9 and C10 chain lengths, conversion was poor. Mono-methyl azelate was clearly a better substrate, since incubations with this substrate yielded 0.50 mM of methyl-ethyl azelate, corresponding to a conversion of 10% of the added mono-methyl azelate. The incubations with mono-ethyl suberate yielded 0.19 mM. These compounds are identical in length, which suggests that the position of the ester group plays a role.

An impurity of di-ester was present in the MEDA stocks; this was confirmed by GC analysis of the pure stock. The impurity was measured at t_0_, this concentration was subtracted to evaluate which share of di-ester was formed by the cells.

### Combining the esterification and ω-oxidation modules

**Fig. 3:**
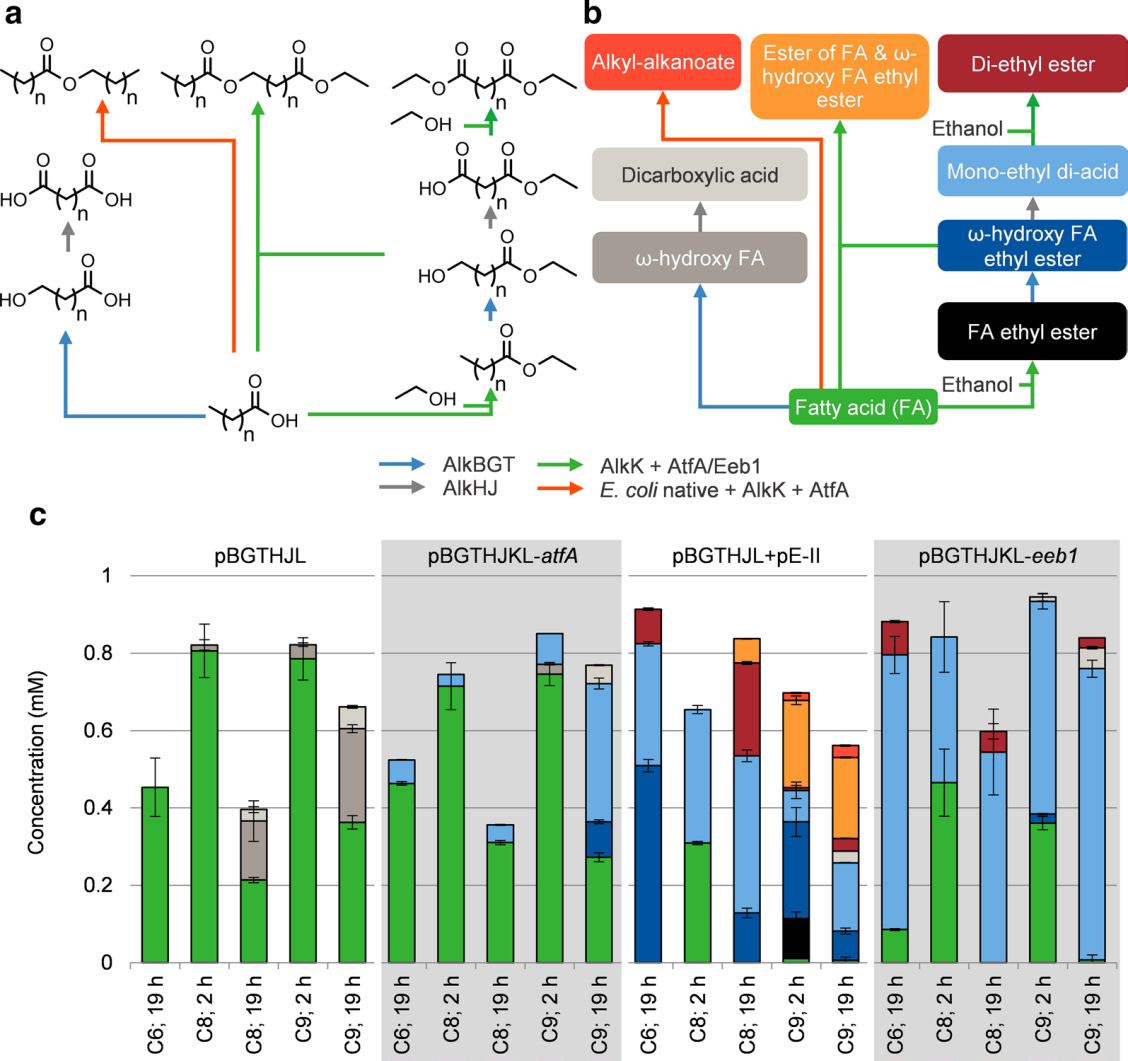
Products detected in whole-cell conversions of different fatty acids. **a** legend indicating the structures of possible products from conversions of fatty acids with resting *E. coli* strains from **c**. **b** the same legend, with the names of the detected compounds. The colors of the boxes correspond with the colors in **c**. The colors of the arrows in **a** and **b** indicate which enzymes catalyze the reactions. **c** resting cell conversion of fatty acids by various *E. coli* strains. Concentration of fatty acid was 1 mM, ethanol was added to 2.5%. C6, C8, C9 represent hexanoic, octanoic and nonanoic acid, respectively. Biomass concentrations were 1.0 g_cdw_/L for all strains

Resting-cell conversions of fatty acids were performed with *E. coli* pBGTHJKL-*atfA*, expressing both modules. The same conversion was done with *E. coli* pBGTHJL, expressing only the ω-oxidation module, to compare the ω-oxidation capacity. The products detected in these tests are shown in Fig. 3a. *E. coli* pBGTHJL produced ω-hydroxy fatty acids and dicarboxylic acids, up to 0.30 mM after 19 h when 1 mM nonanoic acid was the substrate (Fig. 3b). Conversions with hexanoic acid did not yield ω-oxidation products. AlkB thus ω-oxidizes the fatty acids to a limited extent. Apparently, AlkJ and AlkH function poorly with ω-hydroxy fatty acids since the highest titer of dicarboxylic acid was 0.06 mM.

*Escherichia coli* pBGTHJKL-*atfA* produced the ethyl esters of the added fatty acids (Fig. 3b). These ethyl esters were ω-oxidized, resulting in production of 0.36 mM of mono-ethyl azelate after 19 h. These results show shows that further oxidation to the carboxylic acid is more efficient with ω-hydroxy fatty acid ethyl esters than with ω-hydroxy fatty acids. This is in line with earlier findings, where ethyl esters served as substrate [6]. In contrast to nonanoic acid, hexanoic and octanoic acid were not efficiently converted to MEDA. Conversions with these fatty acids yielded at most 0.06 mM mono-ethyl dicarboxylic acid, less ω-oxidized product than in conversions with *E. coli* pBGTHJL.

In general, a large share of the added fatty acids was not converted into product by *E. coli* pBGTHJKL-*atfA*. Intermediate esterified products did not accumulate in this strain, suggesting that ester synthesis (and not oxidation) limited higher product titers. Since *atfA* was equipped with its own P_alkB_ promoter, expression levels of this gene were high (Additional file 1: Figure S1). On the contrary, AlkK is the penultimate gene in the *alk* operon, which resulted in low expression levels. In order to increase the titers, we switched the *ori* and resistance marker from pE to give pE-II, a plasmid that can be cotransformed with pBGTHJL. On this plasmid, AlkK has its own P_alkB_ promoter and could potentially have a higher esterification activity. *E. coli* transformed with pE-II produced 0.72 mM ethyl octanoate from 1 mM octanoic acid and thus performed similar to *E. coli* pE (data not shown). This plasmid was cotransformed with pBGTHJL, giving *E. coli* pBGTHJL + pE-II, which has higher expression levels of AlkK as indicated by SDS PAGE analysis (Additional file 1: Figure S1).

The resulting strain clearly produced more ω-oxidized products, especially with hexanoic and octanoic acid as substrate (Fig. 3b). In the tests with hexanoic acid, 0.91 mM of ω-oxidized product was formed in 19 h. This strain thus had a higher esterification capacity and as a result also further converted MEDAs to di-ethyl esters. Di-ethyl esters were formed from all the tested fatty acids, indicating that the product went twice through the esterification pathway (constituted by AlkK and AtfA). The highest concentration of di-ethyl ester (0.24 mM, 24% molar yield) was detected in conversions with octanoic acid. GC–MS analysis demonstrated that *E. coli* pBGTHJL + pE-II also produced side-products, the major one being the ester of nonanoic acid and 9-hydroxy ethyl nonanoate, or ethyl 9-(nonanoyloxy)nonanoate (see Additional file 1: Figure S2 for proposed pathway). Since this chemical was not commercially available, the quantification was realized by taking the response factor of dimethyl octadecanedioate. Furthermore, nonyl nonanoate was produced, which is probably the result of nonanoic acid reduction to 1-nonanol. This product is then esterified with nonanoyl-CoA to give nonyl nonanoate. Ethyl esters of *E. coli* native fatty acids were also detected, including: ethyl myristate, ethyl palmitate and ethyl oleate. These findings highlight the nonspecific nature of AtfA.

### Investigating di-ethyl ester production

**Fig. 4:**
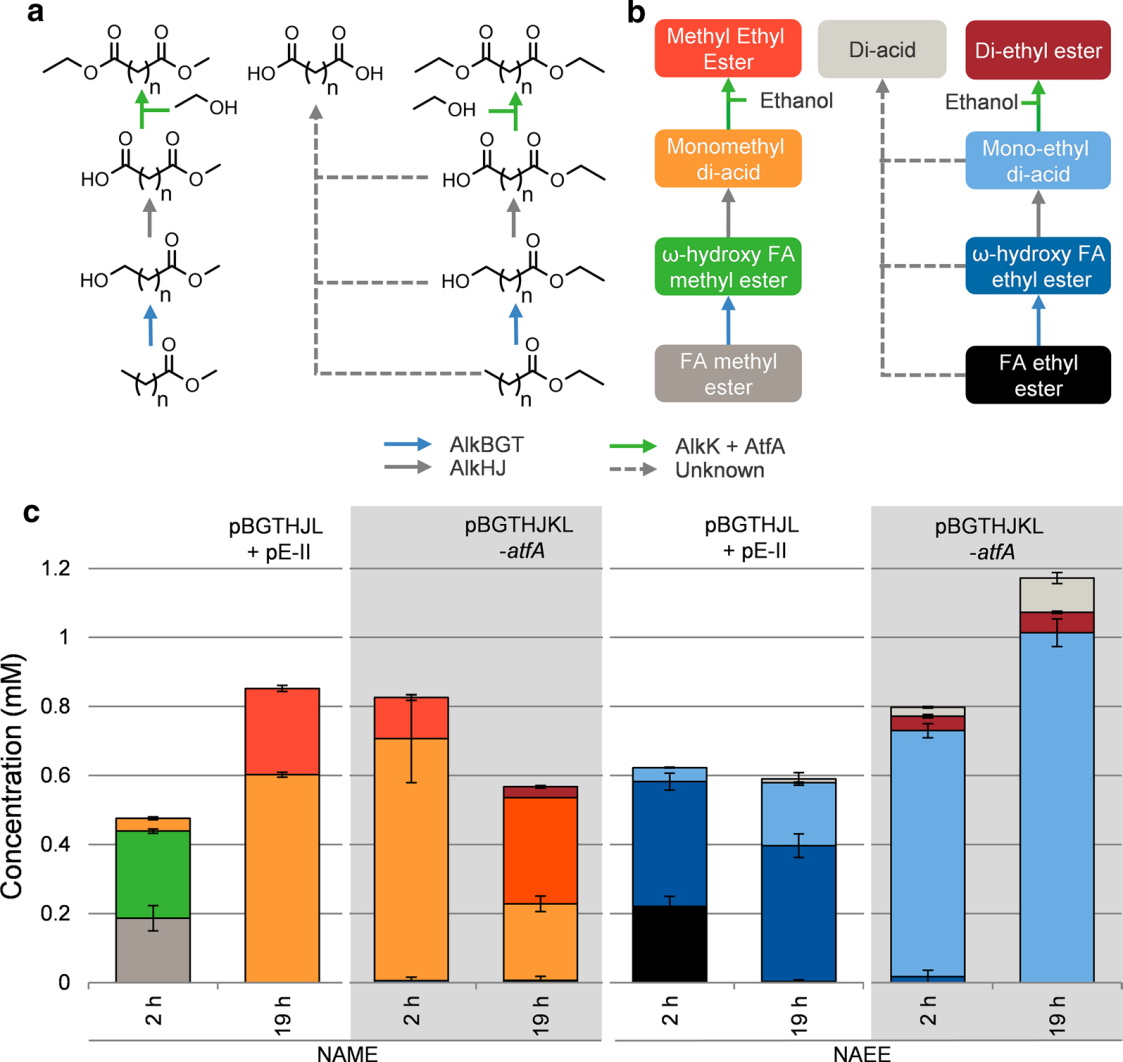
Products detected in whole-cell conversions of methyl nonanoate and ethyl nonanoate. **a** legend indicating the structures of possible products from conversions of fatty acid methyl/ethyl esters with resting *E. coli* strains from **c**. **b** the same legend, with the names of the detected compounds. The colors of the boxes correspond with the colors in **c** The colors of the arrows in **a** and **b** indicate which enzymes catalyze the reactions. **c** resting cell conversions of 1 mM methyl nonanoate (NAME) or 1 mM ethyl nonanoate (NAEE) by various *E. coli* strains. Ethanol was added to 2.5%. Biomass concentrations were 1.0 g_cdw_/L for both strains

Di-ethyl ester production was less efficient than ethyl ester production. It seemed to occur when the fatty acids were depleted, suggesting that fatty acids are preferred over MEDAs as substrate by the esterification module. We therefore checked if di-ethyl ester production could be enhanced, by using ethyl nonanoate instead of nonanoic acid as substrate. Ethyl nonanoate is efficiently converted to the ω-acid by *E. coli* pBGTHJL [6]. The results are shown in Fig. 4.

In these tests, the di-ethyl azelate concentrations remained low as well, whereas mono-ethyl azelate was efficiently produced. This finding suggests that the substrate specificity of AlkK and AtfA causes the low titers. The product distribution of the conversions with *E. coli* pBGTHJL + pE-II and *E. coli* pBGTHJKL-*atfA* were clearly different. The pBGTHJL + pE-II strain has a high esterification activity, but a rather low ω-oxidation activity. The opposite is true for the pBGTHJKL-*atfA* strain. Since the P_alkB_ promoter is strong, the ability to synthesize proteins likely becomes limiting, which can cause the different behavior of the two strains. Since mono-methyl azelate seemed a better substrate for di-ester production (Table 2), methyl nonanoate was added to the same strains, see Fig. 4.

In these tests, methyl-ethyl azelate was formed. The di-ester titers were clearly higher; the highest titer of 0.31 mM was reached by *E. coli* pBGTHJKL-*atfA.* This strain produced a limited amount of di-ethyl azelate as well, indicating that methyl ester hydrolysis had occurred, after which the molecule was esterified with ethanol. After 2 h of incubation, the product distribution in tests with *E. coli* pBGTHJL + pE-II was comparable for both methyl nonanoate and ethyl nonanoate. After 19 h however, the ω-hydroxy fatty acid ester was completely converted to ω-acid in the case of methyl nonanoate, which was not the case for the substrate ethyl nonanoate. This result suggests that the oxygenates of the methyl ester are more efficiently further oxidized to the acid than those of the ethyl ester.

### Improving selectivity with a more selective alcohol-acyltransferase

AtfA prefers longer alcohol and acyl-CoA chain lengths. It is also very nonspecific, which caused the accumulation of by-products as described above. Therefore, we switched to Eeb1 as an alcohol-acyltransferase to investigate whether the production of MEDA and potentially DEDA from fatty acids could be increased (Fig. 3). *E. coli* pBGTHJKL-*eeb1* clearly produced more MEDA. In the conversions with nonanoic acid the highest amount of products accumulated (0.83 mM), with a 75% molar yield of MEDA. Furthermore, 93% of the product was efficiently ω-oxidized to the carboxylic acid. This strain was also more efficient, since more product was synthesized after 2 h of incubation compared to conversions with other strains. DEDA concentrations were not significantly increased.

## Discussion

Fatty acids were poorly oxidized by *E. coli* pBGTHJL. The highest concentration of ω-oxidized product was 0.30 mM after 19 h, with nonanoic acid as substrate. We show that this problem can be solved by first converting the fatty acids into ethyl esters.

We achieved medium-chain ethyl ester synthesis by using *E. coli* that expresses AlkK and AtfA or Eeb1. In vivo esterification of fatty acids results in products with a low solubility, which can facilitate product removal and product inhibition [7, 18, 19]. Highest titer of ethyl ester was reached when nonanoic acid was applied as substrate, resulting in 0.82 mM of ethyl nonanoate from 1 mM nonanoic acid. AlkK also accepts mono-esterified dicarboxylic acids as substrate, which facilitated di-ester production.

This in vivo esterification was combined with ω-oxidation by AlkBGTHJL to form mono-ethyl DCAs with a chain length of C6 to C10. Most successful was the conversion of 1 mM nonanoic acid by *E. coli* pBGTHJKL-*eeb1,* which produced 0.75 mM of mono-ethyl azelate. These titers are in the same order of magnitude as medium-chain dicarboxylic acid production from fatty acids in shake flask experiments reported before [20–22]. To our knowledge however, this is the first report of the combination of ester biosynthesis and ω-oxidation. ω-Oxidation was also achieved without esterification, but this process only yielded 0.30 mM ω-oxidized product. This process was also much slower than the process with esterification. The major products of ω-oxidation of fatty acids were ω-hydroxy fatty acids, even in presence of dehydrogenases AlkJ and AlkH. ω-hydroxy fatty acids are not further oxidized by AlkB [20]. The limited amount of dicarboxylic acids in these tests are thus most likely products of dehydrogenase action, but this process is far more efficient with esterified ω-hydroxy fatty acids. Esterification is thus a promising tool to enhance ω-oxidation of medium-chain fatty acids. Furthermore, there would be no need for esterification of the fatty acid prior to the conversion process, potentially increasing the cost-competitiveness.

Also α,ω-di-ethyl esters accumulated. Biocatalytic wax di-ester production has been reported before [10], but this study is the first to report on biocatalytic di-ethyl ester production. The highest α,ω-di-ethyl ester concentration was 0.24 mM (55 mg/L), which was reached by *E. coli* pBGTHJL + pE-II in conversions with octanoic acid. Production of di-esters was only detectable in the 19 h samples, which suggests that di-ester production only occurs when the fatty acids are depleted and thus no more competition occurs.

Di-ethyl ester synthesis was thus less efficient than ethyl ester synthesis. Since AlkK and AtfA or EEB1 were co-expressed, we were not able to determine which enzyme limits higher product titers. Direct addition of MEDAs to the conversion medium did not yield much more di-ethyl esters. In these test the highest di-ethyl ester concentration (0.19 mM) was reached with mono-ethyl suberate. Using mono-*methyl* azelate as substrate resulted in 2.6-fold higher product titers (0.50 mM), which suggests that the position of the ester moiety plays an important role. Similar differences were seen between conversions with methyl and ethyl nonanoate, since in the conversions with methyl nonanoate clearly more di-ester was produced, 0.34 mM from methyl nonanoate versus 0.04 mM from ethyl nonanoate. Production of a symmetric di-ester would however be more favorable, if the di-ester product is used for a polymerization reaction.

Besides the di-ethyl esters, also byproducts such as nonyl-nonanoate and ethyl 9-(nonanoyloxy)nonanoate were detected in nonanoic acid conversions with *E. coli* pBGTHJL + pE-II. For the production of dicarboxylic acid mono-/di-esters EEB1 seems a more promising candidate, since it prefers ethanol as the alcohol donor for the acyltransferase reaction. The use of this enzyme thus results in less byproduct formation.

To our knowledge this is the first report on whole-cell biocatalytic di-ethyl ester production from fatty acids. Di-ethyl ester production has several advantages over dicarboxylic acid production. Firstly, di-ethyl esters have a low solubility, which could facilitate downstream processing. Secondly, di-ethyl esters can be applied directly as lubricants or plasticizers. Furthermore, if di-ethyl esters are polymerized with an alcohols or amines, ethanol is released from the reaction. If a dicarboxylic acid is used, water is released. Ethanol is easier to remove from the polymerization than water, and can be recycled in the di-ethyl ester synthesis. Unfortunately, the di-ethyl ester concentrations were lower than mono-ester concentrations. Hence, if one wants to improve the di-ester concentration, the specificity of the esterification enzymes AlkK and AtfA/EEB1 has to be improved. Application of an organic phase for in situ product removal could also facilitate higher product titers by shifting the equilibrium more to the product side. It could also alleviate potential toxicity/product inhibition issues.

## Additional file

**Additional file 1: Table S1.** Primers used in this study. **Figure S1.** SDS PAGE analysis of non-induced (-) and DCPK-induced (+) *E. coli* carrying: pBGTHJL + pE-II (lane 1 and 2), pBGTHJKL-atfA (lane 3 and 4). White arrows indicate AlkK, yellow arrows indicate AtfA. **Figure S2.** Proposed pathway for ethyl 9-(nonanoyloxy)nonanoate production.

## References

[1] Metzger JO (2009). Fats and oils as renewable feedstock for chemistry. Eur J Lipid Sci Technol.

[2] Picataggio S, Rohrer T, Deanda K, Lanning D, Reynolds R, Mielenz J (1992). Metabolic engineering of *Candida Tropicalis* for the production of long–chain dicarboxylic acids. Nat Biotechnol.

[3] Beardslee T, Picataggio S (2012). Bio-based adipic acid from renewable oils. Lipid Technol.

[4] Schrewe M, Julsing MK, Lange K, Czarnotta E, Schmid A, Bühler B (2014). Reaction and catalyst engineering to exploit kinetically controlled whole-cell multistep biocatalysis for terminal FAME oxyfunctionalization. Biotechnol Bioeng.

[5] van Nuland YM, Eggink G, Weusthuis RA (2016). Application of AlkBGT and AlkL from *Pseudomonas putida* GPo1 for selective alkyl ester ω-oxyfunctionalization in *Escherichia coli*. Appl Environ Microbiol.

[6] van Nuland YM, de Vogel FA, Eggink G, Weusthuis RA (2017). Expansion of the ω-oxidation system AlkBGTL of *Pseudomonas putida* GPo1 with AlkJ and AlkH results in exclusive mono-esterified dicarboxylic acid production in *E.* *coli*. Microb Biotechnol.

[7] Steen EJ, Kang Y, Bokinsky G, Hu Z, Schirmer A, McClure A (2010). Microbial production of fatty-acid-derived fuels and chemicals from plant biomass. Nature.

[8] Kalscheuer R, Stölting T, Steinbüchel A. Microdiesel: *Escherichia coli* engineered for fuel production. Microbiology. 2006; 152:2529–36. http://mic.microbiologyresearch.org/content/journal/micro/10.1099/mic.0.29028-0. 10.1099/mic.0.29028-0 16946248

[9] Saerens SMG, Verstrepen KJ, Van Laere SDM, Voet ARD, Van Dijck P, Delvaux FR (2006). The *Saccharomyces cerevisiae* EHT1 and EEB1 genes encode novel enzymes with medium-chain fatty acid ethyl ester synthesis and hydrolysis capacity. J Biol Chem.

[10] Kalscheuer R, Uthoff S, Luftmann H, Steinbüchel A (2003). In vitro and in vivo biosynthesis of wax diesters by an unspecific bifunctional wax ester synthase/acyl-CoA:diacylglycerol acyltransferase from *Acinetobacter calcoaceticus* ADP1. Eur J Lipid Sci Technol.

[11] Uthoff S, Stöveken T, Weber N, Vosmann K, Klein E, Kalscheuer R (2005). Thio wax ester biosynthesis utilizing the unspecific bifunctional wax ester synthase/acyl coenzyme a:diacylglycerol acyltransferase of *Acinetobacter* sp. Strain ADP1. Appl Environ Microbiol.

[12] Stöveken T, Steinbüchel A (2008). Bacterial Acyltransferases as an alternative for lipase-catalyzed acylation for the production of oleochemicals and fuels. Angew Chemie Int Ed.

[13] van Beilen JB, Eggink G, Enequist H, Bos R, Witholt B (1992). DNA sequence determination and functional characterization of the OCT-plasmid-encoded alkJKL genes of *Pseudomonas oleovorans*. Mol Microbiol.

[14] Satoh Y, Murakami F, Tajima K, Munekata M (2005). Enzymatic synthesis of poly(3-hydroxybutyrate-co-4-hydroxybutyrate) with CoA recycling using polyhydroxyalkanoate synthase and acyl-CoA synthetase. J Biosci Bioeng.

[15] Jiang Y, Woortman AJJ, Alberda van Ekenstein GOR, Loos K (2015). Environmentally benign synthesis of saturated and unsaturated aliphatic polyesters via enzymatic polymerization of biobased monomers derived from renewable resources. Polym Chem.

[16] Eggink G, Lageveen RG, Altenburg B, Witholt B. Controlled and functional expression of the *Pseudomonas oleovorans* alkane utilizing system in *Pseudomonas putida* and *Escherichia coli*. J Biol Chem. 1987; 262:17712–8. http://www.jbc.org/content/262/36/17712.abstract. 2826430

[17] Julsing MK, Schrewe M, Cornelissen S, Hermann I, Schmid A, Bühler B (2012). Outer membrane protein AlkL boosts biocatalytic oxyfunctionalization of hydrophobic substrates in *Escherichia coli*. Appl Environ Microbiol.

[18] Xin F, Basu A, Yang K-L, He J (2016). Strategies for production of butanol and butyl-butyrate through lipase-catalyzed esterification. Bioresour Technol.

[19] Rodriguez GM, Tashiro Y, Atsumi S (2014). Expanding ester biosynthesis in *Escherichia coli*. Nat Chem Biol.

[20] Clomburg JM, Blankschien MD, Vick JE, Chou A, Kim S, Gonzalez R (2015). Integrated engineering of β-oxidation reversal and ω-oxidation pathways for the synthesis of medium chain ω-functionalized carboxylic acids. Metab Eng.

[21] Kirtz M, Klebensberger J, Otte KB, Richter SM, Hauer B (2016). Production of ω-hydroxy octanoic acid with *Escherichia coli*. J Biotechnol.

[22] Scheps D, Honda Malca S, Richter SM, Marisch K, Nestl BM, Hauer B (2013). Synthesis of ω-hydroxy dodecanoic acid based on an engineered CYP153A fusion construct. Microb Biotechnol.

