# Supplementary material for: Combination of ester biosynthesis and ω-oxidation for production of mono-ethyl dicarboxylic acids and di-ethyl esters in a whole-cell biocatalytic setup with Escherichia coli
Source: Microb Cell Fact. 2017 Nov 2;16:185. doi: 10.1186/s12934-017-0803-9 (PMC5667465; doi:10.1186/s12934-017-0803-9)
Supplement: Supplementary file 1 — Additional file 1: Table S1. Primers used in this study. Figure S1. SDS PAGE analysis of non-induced (-) and DCPK-induced (+) E. coli carrying: pBGTHJL + pE-II (lane 1 and 2), pBGTHJKL-atfA (lane 3 and 4). White arrows indicate AlkK, yellow arrows indicate AtfA. Figure S2. Proposed pathway for ethyl 9-(nonanoyloxy)nonanoate production. [file 12934_2017_803_MOESM1_ESM.docx]

# Additional files to ‘Combination of ester biosynthesis and ω-oxidation for production of mono-ethyl dicarboxylic acids and di-ethyl esters in a whole-cell biocatalytic setup with Escherichia coli’

**Youri M. van Nuland^a^ #, Gerrit Eggink^ab^, Ruud A. Weusthuis^a^**

# Vector construction

Primers that were used are shown in supplementary table 1. For PCR reactions, Phusion polymerase (ThermoFisher Scientific) was used. *alkK* and *atfA* were codon optimized for *E. coli* and synthesised by GenScript^®^. These genes were delivered in pUC57 vectors. Codon optimized *alkK* was amplified from pUC57-*alkKcodopt* using primers 1 and 2. The product and pET-Duet™-1 (Novagen- EMD Millipore) were both digested with NdeI and XhoI; ligation resulted in generation of pET-Duet-*alkK*.
Vector pBGTHJKL-*atfA* was constructed by a Golden Gate approach. pCOM10 was used as backbone, therefore pCOM10-*alkL* was digested with EcoRI and SalI. Primers were designed to generate three inserts, which were *alkBFGHJKL*, P_alkB_ and codon optimized *atfA.* *alkBFGHJKL* was amplified from pGEc47 with primers 3 and 4, P_alkB_ from pGEc47 with primers 5 and 6, codon optimized *atfA* from pUC57-*atfAcodopt* with primers 7 and 8. This PCR yielded three amplicons that were digested with restriction enzymes that are listed in Table 2 and ligated. This strategy enabled ligation in the aforementioned order in pCOM10, which was possible due to the presence of 4 bp overhangs generated from BsaI digestion.

**Table S1. Primers used in this study**

| **Primer #** | **Primer name** | **Sequence** | **Remarks** |
| --- | --- | --- | --- |
| 1 | *alkKcodopt*_fw_NdeI | TAGCTCGC**CATATG**CTGGGTCAAATGATG | NdeI site in bold |
| 2 | *alkKcodopt*_rv_XhoI | GTATCA**CTCGAG**TTATTCACACACCGATGAG | XhoI site in bold |
| 3 | *alkB_*fw_EcoRI | AATTGGA**GAATTC**CATATGCTTGAGAAACACAGAG | EcoRI site in bold |
| 4 | *alkL*_rv_BsaI | CACACCAGGTCTCA**GTTT**TAGAAAACATATGACGCACCAAGACT | overhang in bold, complementary to 5’ P_alkB_ |
| 5 | P_alkB__fw_BsaI | CACACCAGGTCTCA**AAAC**TACCCGTAGGTGTAGTTGGCGCA | overhang in bold complemenary to 3’ *alkL* |
| 6 | P_alkB__rv_BsaI | CACACCAGGTCTCA**ATTC**AGAATTCTCCAATTTTTATTAAATTAGTCG | overhang in bold complemenary to 5’ *atfA* |
| 7 | *atfAcodopt*_fw_BsaI | CACACCAGGTCTCA**GAAT**GCGCCCGCTGCACCC | overhang in bold complemenary to 3’ P_alkB_ |
| 8 | *atfAcodopt*_rv_SalI | GTATCT**GTCGAC**TTAATTTGCCGTTTTGATATCTTC | SalI site in bold |
| 9 | *alkK*_fw_MunI | TATAT**CAATTG**ATGTTAGGTCAGATGATGCGT | MunI site in bold |
| 10 | *atfA*_rv_BamHI | ATAT**GGATCC**TTAATTTGCCGTTTTGATATC | BamHI site in bold |
| 11 | *alkJ*_rv_BsaI | CACACCAGGTCTCA**CACG**TCGCCTCACATTGATGATTTAT | overhang in bold complementary to 5’ *alkL* |
| 12 | *alkL*_fw_BsaI | CACACCAGGTCTCA**CGTG**ATGAGTTTTTCTAATTATAAAGTAATCGCGATG | overhang in bold complementary to 3’ *alkJ* |
| 13 | *alkL*_rv_SalI | ACGC**GTCGAC**CTGCGACAGTGACAGACCTG | SalI site in bold |
| 14 | Amp_fw_BsrGI | TAGTT**TGTACA**GCATGAGATTATCAAAAAGGATC | BsrGI site in bold |
| 15 | pBR322_rv_XhoI | TATA**CTCGAG**ACTCAAAGGCGGTAATACG | XhoI site in bold |

The pBGTHJKL-*atfA* vector was used for the construction of pE. Primers 9 and 10 were used to generate an amplicon from pBGTHJKL-*atfA* that contains *alkK* fused to P_alkB_-*atfA.* This amplicon was digested with MunI and BamHI; backbone pCOM10_*alkL* was digested with EcoRI and BamHI. These two fragments were ligated to give pE. This vector was digested with EcoRI and ligated again, resulting in loss of 987 basepairs of *atfA* at the 5’ end and giving p*alkKL*. pSTBFGHJL was also created by the Golden Gate method. pSTL was used as backbone, which was digested with EcoRI and SalI. *alkBFGHJ* was amplified from pGEc47 with primers 3 and 11. *alkL* was amplified from pGEc47 with primers 12 and 13. These two amplicons were ligated together with the pSTL digest to give pSTBFGHJL. pE was used to generate pE-II, that has a differen *ori* and resistance marker. The pBR322-Amp^R^ cassette from pET-Duet was generated with primers 14 and 15. Both pE and the cassette were digested with BsrGI and XhoI. pE-II was created by ligation of those two fragments.

Figure S1. SDS PAGE analysis of non-induced (-) and DCPK-induced(+) *E. coli* carrying: pBGTHJL + pE-II (lane 1 and 2), pBGTHJKL-*atfA* (lane 3 and 4). White arrows indicate AlkK, yellow arrows indicate AtfA.


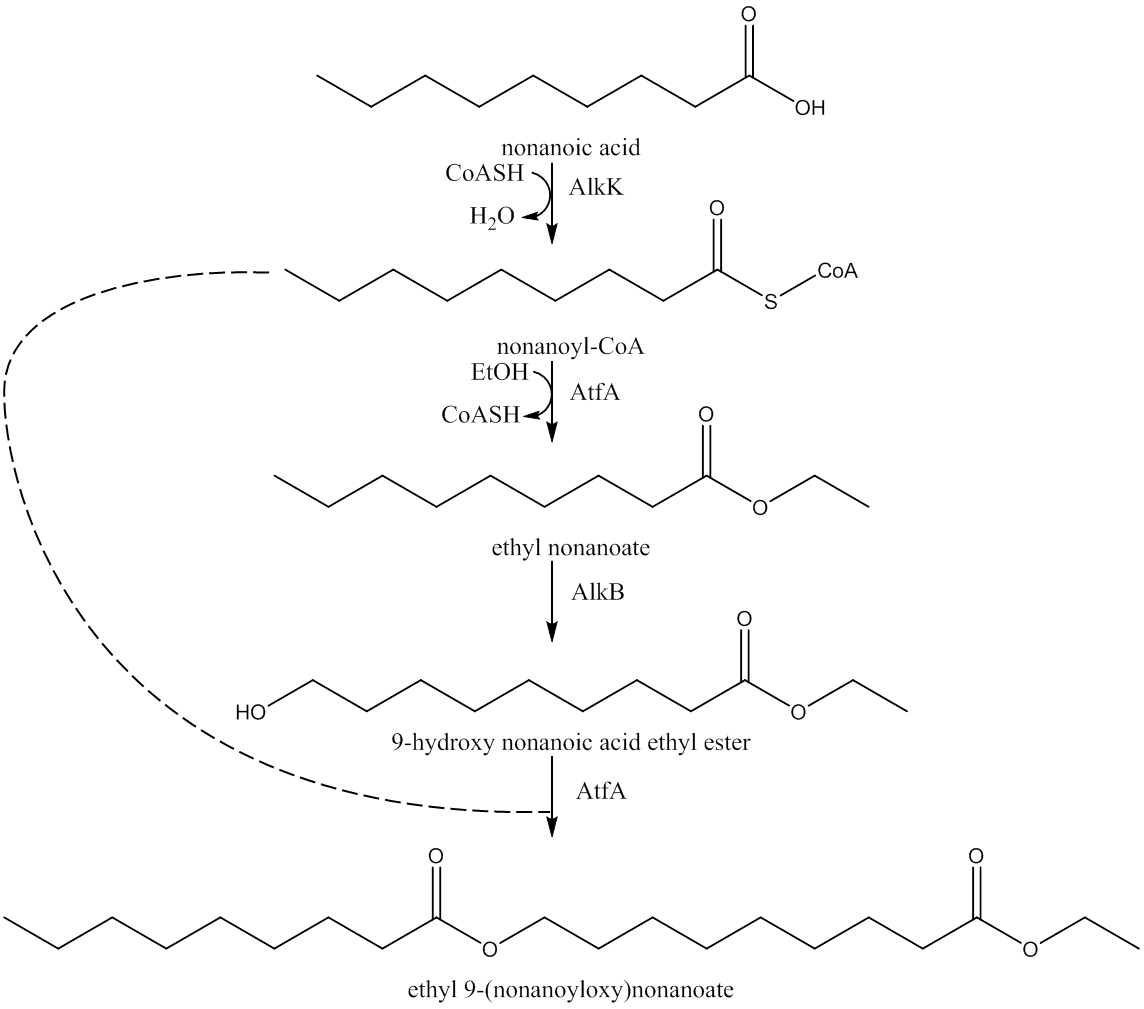


Figure S2. Proposed pathway for ethyl 9-(nonanoyloxy)nonanoate production.
